# An automated system of sample analysis for a total carbon analyser

**DOI:** 10.1155/S146392468400016X

**Published:** 1984

**Authors:** L. Fitzgerald, J. R. Montgomery, J. K. Holt

**Affiliations:** 1 Harbor Branch Foundation R.R. 1 Box 196 Ft. Pierce FL 33450 USA; 2 Center for Marine Biotechnology R.R. 1 Box 196 Ft. Pierce Florida 33450 USA


Journal of Automatic Chemistry, Volume 6, Number 2 (April–June 1984), pages 80–83

### An automated system of sample analysis for a total carbon analyser

**L. Fitzgerald, J. R. Montgomery and J. K. Holt***

*Harbor Branch Foundation, R.R. 1, Box 196, Ft. Pierce, FL 33450, USA*

### Introduction

Standard analysis of the dissolved organic carbon (DOC) content of seawater involves the wet oxidation technique described by Menzel and Vaccaro [1]. In this method the sample is sealed in ampoules containing potassium persulphate. As the samples are heated in a sealed pressure vessel, the organic carbon is oxidized to $CO_2$ which is then measured with a non-dispersive infra-red analyser. Wet oxidation is best suited to seawater analysis because it is unaffected by high salt concentrations and has a high sensitivity.

Due to the large number of samples generated in our research of estuarine and oceanic waters, a fast, economical, automated sample vial handling device was needed which could be used in conjunction with an Oceanography International 0524B Total Carbon System. Our present method of DOC analysis requires tedious and time-consuming manipulation of sealed ampoules and constant operator attention [2]. The automated sample handling system allows the operator to perform other tasks while samples are analysed automatically and with greater efficiency.

Other automated analysis systems for organic carbon have been developed, but they involve photo-oxidation and would require complicated changes in the preparation and analysis of our samples [3 and 4]. Therefore, we decided to automate our present manual system.

This new system uses simple, consecutive logic and a series of limit switches and time delay relays to rotate the ampoule into position, break the top of the glass ampoule and purge each sample of $CO_2$ with nitrogen. We decided that it must be designed with as little alteration of the original instrument as possible and must be able to convert between the manual and automatic mode quickly and easily.

Sets of standard curves were analysed on both the manual and automated systems. The curves were tested for homogeneity as a group using analysis of covariance as described by Zar [5] and Sokal and Rholf [6]. The analysis revealed that there was no significant difference between the regression lines of the standard curves analysed by the different systems although there is an inherent difference caused by variations in the preparation of the samples. Sets of identical samples were also analysed using both methods, and an increase in precision was noted in the automated analysis over the manual method.

### Equipment and methods

The automated system uses sequenced, mechanically-controlled steps for ampoule manipulation and a microprocessor data collection system to record data output. Ampoule manipulation and sample processing are shown in figure 1. As power is applied to the system, limit switches 1, 3 and 5 (LS-1, LS-3, LS-5) are activated. These are located at the retracted position of each air cylinder piston. The air cylinders move certain mechanical parts.

In order to index a sample into position beneath the analysis chamber (figure 2), a solenoid valve diverts compressed air to air cylinder 1 (CL-1). CL-1 extends a piston which catches the toothed wheel on the turning assembly, causing it to rotate. The first vial rotates one position and another vial from the gravity feed chute falls into the next slot. As air cylinder 1 extends, LS-1 opens and LS-2 closes. If the sample is not in the correct

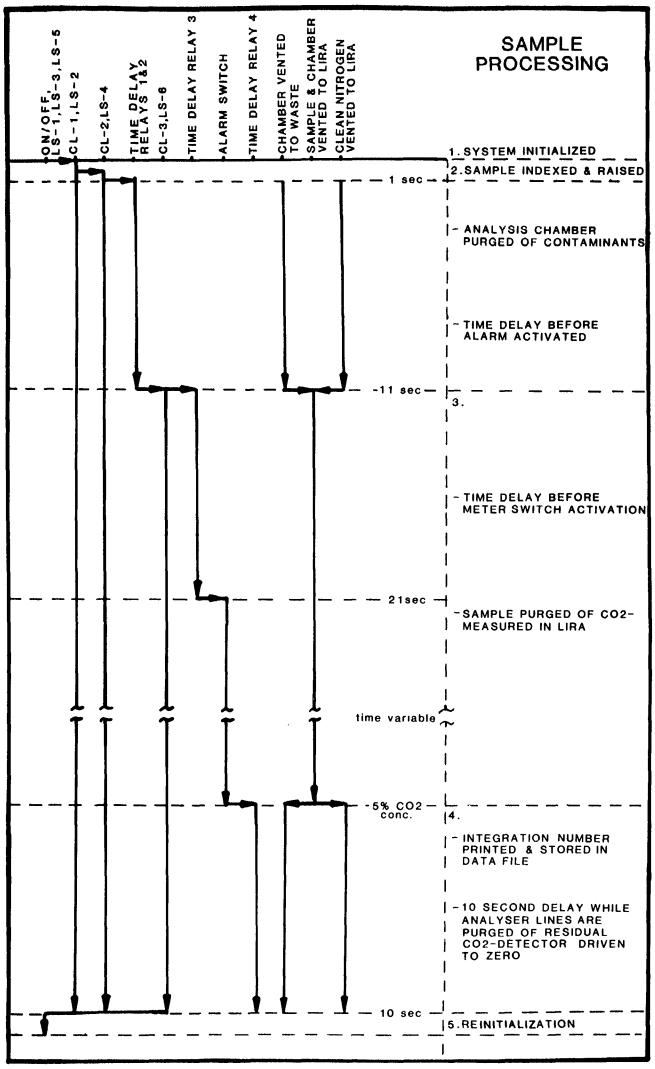

*Figure 1. Flow diagram of sample processing in the automated sample handling device. Horizontal arrows indicate sequential steps. Vertical arrows indicate the mechanisms activated for a period of time.*

* *Present address: Center for Marine Biotechnology, R.R. 1, Box 196, Ft. Pierce, Florida 33450, USA.*

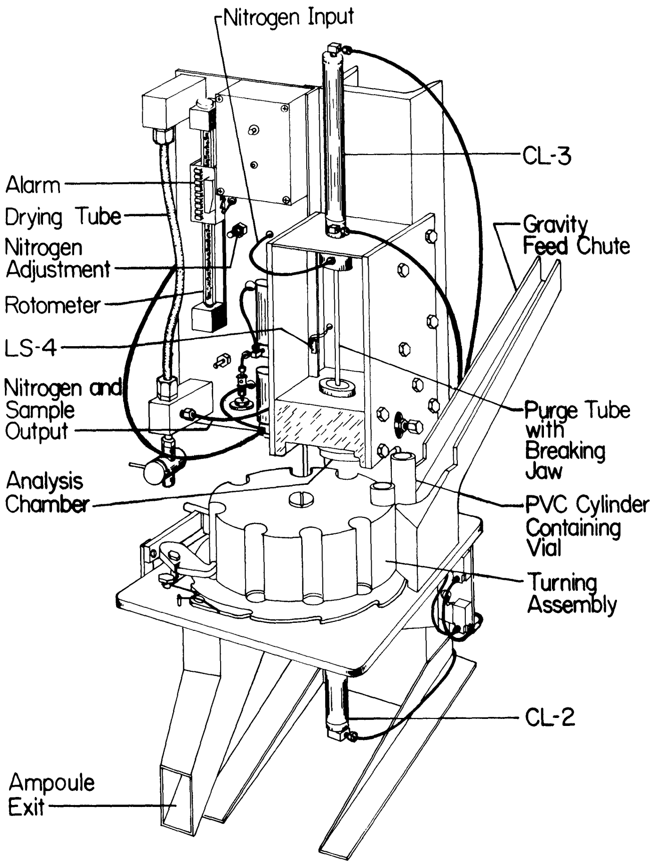

*Figure 2. Diagram of the automated sample handling device. CL-1, LS-1 and LS-2 are located to the left of the turning assembly, but are not visible in this diagram. LS-3, LS-5 and LS-6 are also not visible.*

position, so that CL-1 is not completely extended, then LS-2 does not become activated, and the process stops. This prevents a malfunctioning machine from continuing its cycle and damaging samples.

In the next step a similar process is used to raise the ampoule against the analysis chamber to form the seal. Compressed air is diverted to CL-2 which raises the pedestal on which the ampoule is sitting. The pressure of the gum rubber seal on the ampoule against the walls of the analysis chamber forms a tight seal that prevents gases from escaping. At this point LS-3 is open and LS-4 is closed. The time span for implementation of the first two steps is about one second.

After the analysis chamber is closed by the ampoule seal, two time delay relays (1 and 2) are activated simultaneously. One relay allows 10 s for the chamber to be purged of contaminants and the other delays activation of the photoelectric proximity switch on the flow rate alarm. The alarm is needed to warn the operator when the nitrogen flow rate has deviated from 200 ml/min. The time delay is necessary so that the initial instability of the flow rate will not set the alarm off. If the flow rate is correct, compressed air is diverted to CL-3; this forces the plunger down, breaking the top of the ampoule; LS-5 opens, LS-6 closes and nitrogen purges the chamber and ampoule before flowing to the Lira Infrared Analyser. As the meter needle deflects above zero, a third 10 s time-delay relay (3) allows the needle to pass the proximity switch at 5% on the meter before this switch is activated. Therefore, the switch will sense the meter needle only on its way back down when the sample has been purged of $CO_2$.

When the Lira Analyser detects a $CO_2$ concentration of 5% in the purge gas, the R-F proximity switch senses the meter needle and activates a 10 s delay (4). During this time, the nitrogen flow is diverted to 'waste' to purge the analyser lines of residual $CO_2$. The integrated value for the sample is printed and stored in the computer. After 10 s, all relays and switches are de-energized and all air cylinders retract to their original positions reactivating LS-1, LS-3 and LS-5. The process begins again with the next sample. The data are sent to a microprocessor which creates a file so that it may be accessed later by the data reduction software.

The present working model is the result of many changes in the preliminary design. Special PVC casings and ampoule seals had to be designed which would protect the ampoule from breakage during processing and provide an adequate seal during analysis. After testing many prototypes, a separate 3/16 in wall, 5/16 in i.d. seal is placed on the neck of each ampoule. The ampoule is inserted into 13/16 in i.d. PVC cylinder and placed in the gravity feed chute.

The breaking jaw and purge tube have been combined in the new system to simplify analysis. The purge tube is used to bubble the nitrogen through the sample. It has been made larger and notched on the end to facilitate breakage of the ampoule tip when the plunger comes down. On the plunger's upward stroke, after analysis, the air contained in air cylinder 3 is blown out through two holes just outside the analysis chamber. This expels fragments of glass which may interfere with the ampoule seal.

Since flow rate is crucial when determining concentration, an alarm was installed which would alert the operator of variations in the nitrogen flow. The ball of a rotometer breaks the beam of a photoelectric switch when the flow rate is correct. If the flow is greater or less than 200 ml/min, the light beam is unbroken, the circuit closes and the alarm sounds.

Two gas connections, which may easily be disconnected in conversion between the two modes, run between the manual and automated units. One line splits at the end and connects at the zero gas (nitrogen) and span gas (300 ppm $CO_2$) valves on the manual unit. The other is the output line to the Lira Analyser. Electrical connections run to the meter and to the print circuit of the integrator on the manual unit.

### Results

Two series of standard curves were analysed to test the accuracy and precision of the automated system. Six standard curves were run on the automated system while seven were run manually for comparison. Each curve consisted of four to six standard concentrations. Linear regressions were computed for each standard curve and the regression coefficients, correlation coefficients, $f$-ratios and $y$-intercepts are shown in table 1.

An analysis of covariance (ANCOVA) was used to determine if there was any statistical difference between the slopes of the linear regressions analysed on the two systems. The ANCOVA tests the linear regressions to determine if automation contributes added variability to the data set. First, the ANCOVA tests the slopes of each regression to see if they could have come from populations with the same slope. Then a common regression line is computed using the means of $Y$ and tested for heterogeneity of the means about this line. Finally the common regression is compared to a pooled regression of all the $XY$ pairs [6].

To test if the linear regressions have similar slopes, a variance ratio, $F$, was computed and compared to critical values of the $F$-distribution at the 5% probability level using K-1 and the pooled residual degrees of freedom ($F$ 0·05, 12, 230) [5]. The calculated

*Table 1. Statistical data for linear regressions used in the analysis of covariance (ANCOVA).*

| Regression No. | $N$ | Regression coefficient | $Y$ intercept | Residual sum of squares | Residual D.F. | $F$ ratio | Correlation coefficient |
|---|---|---|---|---|---|---|---|
| 1 | 10 | 368537 | −90·8 | 423 872 | 8 | 974 | 0·996 |
| 2 | 13 | 342315 | 775·4 | 144 162 | 11 | 1868 | 0·997 |
| 3 | 16 | 386807 | −41·7 | 1190 970 | 14 | 963 | 0·993 |
| 4 | 18 | 397054 | 7·8 | 158 048 | 16 | 10439 | 0·999 |
| 5 | 16 | 364727 | −84·1 | 396 256 | 14 | 2301 | 0·997 |
| 6 | 18 | 351015 | −12·7 | 249 600 | 16 | 5165 | 0·999 |
| 7 | 27 | 394871 | 393·9 | 606 656 | 25 | 6257 | 0·998 |
| 8 | 26 | 411311 | 125·4 | 666 784 | 24 | 5823 | 0·998 |
| 9 | 27 | 342599 | −112·1 | 3092 240 | 25 | 915 | 0·987 |
| 10 | 25 | 332773 | 81·2 | 2359 020 | 23 | 1030 | 0·989 |
| 11 | 21 | 325912 | 143·3 | 215 312 | 19 | 6397 | 0·999 |
| 12 | 11 | 360822 | 13·9 | 1299 380 | 9 | 205 | 0·985 |
| 13 | 28 | 379593 | 45·1 | 264 208 | 26 | 15017 | 0·999 |
| Pooled | | | | 11 066 500 | 230 | | |
| Common | | 368239 | 95·7 | 17 808 500 | | | |
| Total | 256 | | | 50 427 400 | 254 | | |

$F$ was 11·68 with a critical value between 1·83 and 1·75, therefore the null hypothesis that B1 = B2 = ... = B$m$ was rejected. This means the regression coefficients were significantly different within the total group of automated and manual curves and the standard curves were not from populations with the same slope. The standard curves of both systems were analysed separately for analysis of covariance. For the manual system, the calculated $F$ was 16·79 with a critical $F$ (0·05, 6, 151) between 2·10 and 2·17. The ANCOVA for the automated system calculated an $F$ of 6·37 with a critical $F$ (0·05, 5, 79) between 2·29 and 2·37. In both cases, the ANCOVA rejected the standard curves as coming from populations of the same slope. Therefore, the linear regressions within the groups for each system did not have similar slopes.

Our next step was to determine which slopes were different, and whether the difference was due to automation. The Newman–Keuls multiple range test compares each combination of two regression lines to determine whether they are similar. When two regressions are compared, a test statistic $q$ is calculated using the slope of each regression and the standard error [5]. This statistic was compared to the critical $q$ (0·05, 230, $p$), where $p$ is the number of regressions being tested and 230 was the pooled degrees of freedom. Each slope was compared to every other slope and the results are reported in table 2. The regressions have been ranked by decreasing slope and form five overlapping groups. Within each group the slopes were determined to be statistically similar. Note that within each group there are both manually and automatically analysed standard curves.

The relative standard deviation (RSD) was computed for the replicates of each standard concentration in each standard curve. The sets of replicates analysed on the manual system indicated a mean RSD of 4·4%, while the analyses of replicates from the automated system gave a 4·3% mean RSD. After the 33 sets of replicates tested on the automated system were analysed, the method of sample digestion was changed from a heated pressure vessel to a boiling water bath. A total of 204 sets of replicates was analysed using the water bath digestion procedure. When all the sets of replicates analysed on the automated system were taken into account, the mean RSD was 3·7%. This increase in precision was probably due to the improvements made in the system with time, and not the method of digestion. This change in digestion procedure had no effect on the precision of analysis because it only affected the extent of digestion. Therefore, sets of replicates digested at the same time were digested to the same extent.

A comparison of the accuracy of the two methods was made using Florida Department of Environmental Regulation (DER) performance evaluations for 1981 and 1982. The DER sent two samples of known concentration to participating laboratories to be tested for total organic carbon (TOC). The data for the manual (1981) and automated (1982) systems are shown in table 3. The DER computed 95% and 99% confidence intervals from

*Table 2. The standard curves used in ANCOVA ranked in order of decreasing slope and classified into five overlapping groups of statistically similar regressions by the Newman–Keuls multiple range test.*

| Regression No. | Method of analysis | Regression coefficient | | | | | |
|---|---|---|---|---|---|---|---|
| 8 | Manual | 411311 | | | | | |
| 4 | Auto | 397054 | Group 1 | | | | |
| 7 | Manual | 394871 | | | | | |
| 3 | Auto | 386807 | | | | | |
| 13 | Manual | 379593 | | Group 2 | | | |
| 1 | Auto | 368537 | | | | | |
| 5 | Auto | 364727 | | | Group 3 | | |
| 12 | Manual | 360822 | | | | | |
| 6 | Auto | 351015 | | | | Group 4 | |
| 9 | Manual | 342599 | | | | | |
| 2 | Auto | 342315 | | | | | Group 5 |
| 10 | Manual | 332773 | | | | | |
| 11 | Manual | 325912 | | | | | |

*Table 3. Comparison of the manual and automated systems using data from 1981 and 1982 Florida Department of Environmental Regulation Quality Assurance Program (Jack Merritt, DER, personal communication).*

| | 1981 Manual system | | 1982 Automated system | |
|---|---|---|---|---|
| | Sample 1 | Sample 2 | Sample 1 | Sample 2 |
| Laboratory value (mgC/l) | 13·40 | 27·30 | 6·53 | 15·78 |
| Expected value (mgC/l) | 14·14 | 32·33 | 5·92 | 16·18 |
| Pooled lab. data: | | | | |
| Mean recovery (mgC/l) | 13·771 | 32·138 | 6·2521 | 16·085 |
| Sample size | 19 | 20 | 24 | 25 |
| Standard deviation | 2·1907 | 4·8599 | 0·8815 | 2·4725 |
| Percent RSD | 15·9 | 15·1 | 14·1 | 15·1 |
| 95% Confidence interval | 9·48–18·06 | 22·6–41·7 | 4·52–7·98 | 11·23–20·94 |
| 99% Confidence interval | 8·12–19·42 | 19·6–44·7 | 3·98–8·53 | 9·70–22·47 |

the pooled data of the participating laboratories. Our values for both years were within the 99% confidence intervals.

A chi-square analysis was computed to measure the closeness of agreement between the observed and expected values. The manual system gave a calculated $\chi^2$ value of 10·2654 which indicates no significant difference between the observed and expected values up to approximately the 70% confidence level (where $\chi^2$ 0·75, 14 = 10·165 and $\chi^2$ 0·50, 14 = 13·339). On the other hand, the automated system gave a calculated $\chi^2$ value of 1·1012 which indicates no significant difference at 99·9% confidence level ($\chi^2$ 0·001, 11 = 1·834). Therefore, the automated system was much more accurate than the manual system.

### Discussion

This new method for sample vial handling provides for transport and positioning as well as reliable isolation of the sample from ambient contamination. It also provides precision sampling of a gas by purging, or a liquid by precision pump or other suitable unit. The versatility of this system allows it to purge a gas from any liquid matrix and deliver that gas to any appropriate gas detection system.

The advantages of this system in relation to the Oceanography International 0524B Total Carbon System are numerous. This system requires little operator assistance. It increases productivity by freeing up the operator to do other tasks and by analysing samples much faster than manual methods (from approximately 80 manually to 200 automatically analysed samples per 8 h). Only minor modifications of the manual system are necessary when converting to the automatic mode. The automated system constantly purges the analyser when samples are not being measured for $CO_2$. The manual system vents the nitrogen between samples.

The methods used to test for differences between the regression coefficients of the standard curves analysed with the manual and automated systems gave interesting results. The ANCOVA and Newman–Keuls multiple range tests reported five overlapping groups of standard curves whose regression coefficients within each group were similar. Although the analysis determined that there was a significant difference in the slopes of the standard curves of the automated and manual systems this does not indicate that the difference was due to the method of analysis. The five groups in which the regression coefficients were similar contained both manual and automated curves in each (table 2). This indicates an inherent difference in the method of preparation and digestion of the samples in each standard curve and not in the method of analysis.

Precision improved after the new system was used routinely. In the new system, analysis of each sample is finished at exactly 5% $CO_2$ concentration. This cut-off point is determined visually in the manual system, therefore each sample may not be handled in exactly the same way. This is shown by the difference in precision before and after the new system went into use.

The accuracy was tested during DER performance evaluations. The laboratory values for both samples for both years were in the 99% confidence interval as determined by the DER's evaluation of all participating laboratories. Thus, the automated system is comparable in accuracy to the manual system. The chi-square analysis showed the automated system to be more accurate than the manual one.

The automation of the Total Carbon System is not complete. Conversion to DC power is planned to reduce noise in the system. An improved flowmeter and alarm system will be installed and the present hardwired controller will be replaced with a programmable one.

### Acknowledgements

We would like to thank Gary Peterson and Andrew Clark for their help in the design and modification of the automated system and also for their valued advice in the writing of this paper. This is Harbor Branch Foundation Contribution Number 363.

### References

1. Menzel, D. W. and Vaccaro, R. F., *Limnological Oceanography*, **9** (1964), 138.
2. Peterson, G. N. and Montgomery, J. R., *Harbor Branch Foundation Technical Report*, **38** (1981), 71.
3. Goulden, P. D. and Brooksbank, P., *Analytical Chemistry*, **47** (1975), 1943.
4. Collins, K. J. and Williams, P. J. le B., *Marine Chemistry*, **5**, (1977), 123.
5. Zar, J. H., *Biostatistical Analysis* (Prentice-Hall, Englewood Cliffs, New Jersey, 1974).
6. Sokal, R. R. and Rholf, F. J., *Biometry* (Freeman, San Francisco, 1969).

